# High-Grade Limbs Open Fractures: Time to Find Milestones in the Emergency Setting

**DOI:** 10.3390/life11111226

**Published:** 2021-11-12

**Authors:** Michele Altomare, Stefano Granieri, Stefano Piero Bernardo Cioffi, Andrea Spota, Silvia Azisa Basilicò, Osvaldo Chiara, Stefania Cimbanassi

**Affiliations:** 1 Acute Care Surgery and Trauma Team, ASST Niguarda, Piazza Ospedale Maggiore 3, 20162 Milan, Italy; michele.altomare@ospedaleniguarda.it (M.A.); stefanopiero.cioffi@niguarda.it (S.P.B.C.); andrea.spota@unimi.it (A.S.); osvaldo.chiara@unimi.it (O.C.); 2 Department of Surgical Sciences, Sapienza University of Rome, Piazzale Aldo Moro 5, 00185 Rome, Italy; 3 Vimercate Hospital, Via Santi Cosma e Damiano 10, 20871 Vimercate, Italy; stefano.granieri@asst-brianza.it; 4 General Surgery, ASST Rhodense, Corso Europa 250, 20017 Milan, Italy; silvia.basilico85@gmail.com; 5 Department of Medical-Surgical Physiopathology and Transplantation, University of Milan, Via Festa del Perdono 7, 20122 Milan, Italy

**Keywords:** trauma, Gustilo–Anderson, extremity injuries, Mangled Extremity Severity Score

## Abstract

(1) Background: The Gustilo–Anderson (G/A) grading system is a universally accepted tool used to classify high-grade limb open fractures. The purpose of this study is to find early independent predictors of amputation in emergency settings. (2) Methods: A retrospective analysis involving patients treated at our center between 2010 and 2016 was conducted. Patients with at least one G/A grade III fracture or post-traumatic amputation were included. Three groups were identified: G/A IIIA (A group), G/A IIIB-C (BC group), and Amputation group (AMP group). Each group was further divided into two subgroups considering timing of coverage (early vs. delayed). Univariate and multivariate logistic regression models were developed to identify independent predictors of the limb’s outcome. (3) Results: One-hundred-six patients with G/A III A-B-C fractures or amputation of the affected limb were selected from the Niguarda Hospital Trauma Registry. The patients were divided into the A group (26), BC group (66), and AMP group (14). The rate of infectious complications following early or delayed coverage was evaluated: A group, 9.1% vs. 66.7% (*p* > 0.05); BC group, 32% vs. 63.6% (*p* = 0.03); and AMP group, 22% vs. 18.5% (*p* > 0.05). After further recategorization, the BC subgroups were analyzed: multivariate logistic regression model identified systolic blood pressure (SBP) <90 mmHg (*p* = 0.03) and Mangled Extremity Severity Score MESS ≥ 7 (*p* = 0.001) were determined to be independent predictors of limb amputation. (4) Conclusion: MESS and SBP serve as predictors of amputation. Based on the results, we propose a new management algorithm for mangled extremities. Early coverage is related to lower rates of infectious complications. Referral to high-volume centers with specific expertise is mandatory to guarantee the best results.

## 1. Introduction

In Italy, the mortality rate related to major trauma is 3.8% and peaks at 62.4% considering the 15–30 y/o male population [1]. Open fractures are defined by the presence of a break in the skin with air exposition of the stumps and/or related hematoma [2] and are classified according to the Gustilo–Anderson grading system [3]. This system is based on fracture patterns and on soft tissue-related damage. Increasing the severity of fracture pattern is associated with higher infection rates and failure in bone fragment healing. However, this system is subjected to several limitations related to the outcome of the limb [4,5]. Since its introduction in 1976, multiple modifications have been suggested, and the most recent proposed by the original authors can still be used to optimize communication and initial management of these patients [6]. A recent survey conducted by the Italian Society of Orthopedics and Traumatology (SICOT) research academy highlighted that about 94.7% of orthopedic surgeons around the world use the Gustilo–Anderson scoring system for assessments of open injury [7,8].

The primary aim of this study was to identify predictors of definitive outcome for the affected limb (amputation vs. salvage), including the Gustilo–Anderson grading system. 

The secondary aim was to describe the incidence of infectious complications according to the timing of coverage: early (≤3 days) vs. delayed (>3 days).

## 2. Materials and Methods

This retrospective study was based on data collected from the Trauma Registry at Niguarda Hospital between 1 January, 2010, and 31 December 2016. All patients with at least one Gustilo–Anderson grade III open fracture or post-traumatic amputation proximal to the wrist or ankle were selected, and for each patient, the following data were retrieved: patient history (Allergies, Medication, Past medical history, Last meal, Enviroment—AMPLE and American Society of Anesthesiology—ASA classification); prehospital management; emergency department (ED) procedures; first detection of systolic blood pressure (SBP) and heart rate (HR) at admission; base excess (BE) and lactate (Lac) at first Arterial Blood Gases (ABG) analysis performed in ED; occurrence and grade of injury; surgical interventions, occurrences, and types of infection; Mangled Extremity Severity Score (MESS) [9]; prehospital time; length of stay (LOS); and definitive limb’s outcome (amputation vs. salvage). The patient sample was then stratified according to different grades of injury into three groups: G/A IIIA (A group), G/A IIIB-C (BC group), and Amputation group (AMP group). For each group, we further identified subgroups: early coverage, ≤3 days (E group); delayed coverage, >3 days (D group). The BC group was further divided into three subgroups considering the final outcome of the limb: saving the damaged limb (BC-SAV), amputation within 24 h from arrival in ED (BC-AMPT0), and amputation after 24 h from arrival in ED (BC-AMPT1). The management algorithms proposed are based on an analysis of the outcomes in major trauma patients with open fractures associated with extensive soft tissue damage (Gustilo–Anderson grade III—G/A III) evaluating the Injury Severity Score (ISS), the timing of definitive coverage of soft tissue, the rate of infection, and the definitive outcome of the damaged limb (salvage vs. amputation). A Strengthening the Reporting of Observational Studies in Epidemiology (STROBE) statement and checklist for observational studies were followed during the study planning and execution.

### Statistical Methods

The results are reported as absolute values and percentages for categorical variables and as means ± standard deviation (SD) or medians ± interquartile range (IQR) for continuous variables, based on their distribution. Differences in the proportions were evaluated with χ^2^ or Fisher’s exact test for categorical variables. The distribution of each continuous variable was evaluated with the Shapiro–Wilk test. In addition, ANOVA, the Kruskal–Wallis test for independent samples, and the Mann–Whitney test were performed to evaluate the differences among groups for continuous variables. Among the variables for which a significant difference was observed, systolic blood pressure (SBP) and MESS were identified as being of highest interest. After re-coding them as categorical variables by setting the cutoff levels at 90 mmHg and 7 mmHg, respectively [10,11], they were individually included in a univariate logistic regression model to identify possible predictors of the limb outcome (preserved vs. amputated); odds ratios and 95% CI were provided as well. A *p*-value below 0.05 was considered statistically significant.

## 3. Results

During the study period, 3920 major trauma patients were identified in the Niguarda Hospital Trauma Registry. One-hundred-seventeen patients presenting in the ED with G/A III A-BC fractures or amputation of an affected limb were selected. We excluded two patients from the A group, six patients from the BC group, and three patients from the AMP group who died during recovery in the ICU so information on their final outcomes was not attainable. Thus, the A group was composed of 26 patients (24.5%), the BC group was composed of 66 patients (62.2%), and 14 patients formed the AMP group (13.2%). The median age was 43 ± 18.5. The distribution of the mechanism of trauma, hemodynamic status, and ISS score of the three groups are summarized in Table 1. Once re-coded as categorial variables, SBP and MESS were individually entered in a univariate logistic regression model. Both SBP < 90 mmHg (OR: 3.75; 95% CI: 1.133–12.434; *p* = 0.03) and MESS ≥ 7 (OR: 18.25; 95% CI: 3.581–91.729; *p* < 0.001) resulted in being predictors of limb amputation. Furthermore, analyzing SBP as a continuous variable, the regression model pointed out a 2.1% risk reduction in amputation per mmHg increase in SBP (OR 0.979; 95% CI: 0.962–0.996; *p* = 0.015). Both variables were entered in a multiple regression model and were confirmed to be independent predictors of limb amputation (SBP < 90 mmHg: OR: 4.63, 95% CI: 1.013–21.19, *p* = 0.048; MESS ≥ 7: OR: 13.22, 95% CI: 2.489–70.235, *p* = 0.002). The infection rate in the A group was 14.8% (4), that in BC group was 50% (33), and that in AMP group was 27.3% (4). The comparison of infection rates between the groups was significant (*p* = 0.048). The differences in percentages of early vs. delayed coverage in each group were evaluated: A group, 9.1% vs. 66.7% (*p* > 0.05); BC group, 32% vs. 63.6% (*p* = 0.03); and AMP group, 22% vs. 18.5% (*p* > 0.05). The mean closure time (days) of early and delayed coverage were as follows: A group, 0.41 ± 0.63 vs. 35.3 ± 28.4; BC group, 0.32 ± 0.46 vs. 56.7 ± 41.2; and AMP group, 0.71 ± 1.03 vs. 24 ± 1.4. A comparison between nosocomial and community-acquired infections in each group did not highlight any significant difference. Considering the type of infection, in the A group, all 4 cases were nosocomial infections by multi-drug resistant (MDR) bacteria; in the BC group, of 33 infections, 21 (63.6%) were nosocomial (10 *Acinetobacter Baumanii*, 4 *Pseudomonas Aeruginosa*, 2 *Staphylococcus Aureus* methicillin-resistant, 2 *Klebsiella Pneumoniae* carbapenemase-producing, 1 *Enterobacter Cloacae*, 1 *Staphylococcus Warneri*, and 1 *Corynebacterium Striatum*); and in the AMP group, 2 out of 4 infections were nosocomial (*Acinetobacter Baumanii*). Table 2 summarizes the comparisons among the BC group subgroups considering: systolic blood pressure (SBP), heart rate (HR), shock class over 3 (SC > 3), base excess (BE), lactates (Lac), length of stay in ICU (ICU-LOS), total hospital length of stay (H-LOS), AIS ≥ 3 head–chest–abdomen-associated injuries, G/A IIIC fractures, and MESS.

## 4. Discussion and Conclusions

The initial assessment and management of patients with open fractures of the lower or upper extremities should always follow the ATLS guidelines [12]. In the first hours, these cases could be challenging for trauma surgeons. Current guidelines, supported by the British Orthopedic Association (BOA), suggest that the first surgical procedure should be performed within 6 hours from the original accident [13]. This relies on Friedrich’s historical study on guinea pigs in which he evaluated the replication rate of bacteria and deemed 6 hours as the cut-off after which massive replication of bacteria was detected [14]. Albeit supported by scarce evidence, a 6-hour cut-off has been widely accepted and a recent review of the literature [15], including 10 studies [16,17,18] from past decades, confirmed the lack of evidences. Our study underlines the relevance of early closure of the skin after accurate debridement. Based on the mean closure times observed, we demonstrated how the rate of infection is lower in patients who received early coverage, when feasible, within 24 h from trauma in Gustilo–Anderson Grade III A and B-C. This is in contrast to evidence from a recent systematic review of the literature and metanalysis in which no differences were observed in terms of development of infectious complications. However, when patients presenting with G-A IIIA injuries who underwent single-stage orthoplastic fixation and coverage were compared with those who had multiple reconstructive procedures, a difference was found in favor of single-stage patients [19].

No evident difference was found between nosocomial and community infections. In agreement with other available information in the literature [20,21,22,23,24,25,26], most of the patients in the AMP group received early coverage.

A subgroup analysis was then conducted among the BC group to evaluate the final outcome of the injured limb (salvage vs. amputation) and its relationship with the examined variables. The BC group was divided into three subgroups: BC-SAV, BC-AMPT0, and BC-AMPT1. An interesting outcome of this comparison is a significantly longer hospital and ICU length of stay in the BC-AMPT1 group. This result can be better understood considering the initial attempt at limb rescue followed by amputation due to necrosis (77.8%) or worsening of the hemodynamic condition (22.2%). Attempts to rescue the limb in patients with necrosis have often delayed decisions to proceed with T1 amputation and lead to significant increases in hospitalization times. This also has a negative contribution to the delay in rehabilitation needed for prosthetic replacement of the amputated limb. Interestingly, infections of the affected limb were not involved in the decision for amputation considering that, in the AMP group, the incidence of stump infection does not appear to be statistically different between the early and delayed groups.

Therefore, it is important to identify predictive factors for early amputation in these patients in order to avoid delays, to facilitate return to normal daily life, and to reduce the costs for the national health system. The only parameter that was distributed differently among the three subgroups was the SBP value due to the patient’s peripheral vasoconstriction resulting in rapid changes during hemodynamic deterioration. Another interesting result from the analysis is the lack of correlation between severe associated injuries and outcome of the limb. In the literature, one of the main prognostic factors for early amputation of a T0-injured limb is the presence of severe associated injuries, especially in the cranio-encephalic area [10]. 

The final variable analyzed was MESS, for which a statistically significant difference between the three subgroups was found. When compared with patients of the BC-SALV group, T0- and T1-amputated patients showed remarkably higher values of MESS. Regression models then were performed to assess the ability of SBP and MESS to predict the definitive outcome of the limb (saved vs. amputated). Univariate and multiple regression models showed that both variables are strongly associated with limb’s outcome. More precisely, MESS values ≥ 7 and SBP values < 90 mmHg are strongly correlated with amputation. The correlation between SBP and outcome of the limb is consistent with other studies in the literature. The WTA 2011 plenary paper on management of the mangled extremity as well as the study published by De Mestral et al. (2013) demonstrated how persistent hypoperfusion due to low blood pressure represents one of the main predictors for amputation [10,27,28].

In the literature, even though persistent hypotension is a well-known predictor associated with the need for amputation of mangled extremities, no algorithms were formed including the numerical cut-off guiding subsequent steps in the decision-making process. Hemodynamic stability (or instability) is more frequently used to build algorithms thanks to the freedom of interpretation [28]. Thus, one key point of our article is to identify objective criteria that could guide trauma surgeons in difficult treatments of mangled extremities. From this perspective, SBP < 90 mmHg acts as the first “red flag” that alerts of the possible final outcome of the affected limb. In our study, the strong correlation between MESS and the outcome of the limb contrasts evidence from the literature. In particular, our findings are discordant with the ones reported by Bosse et al. (2001) [11]; the authors indeed showed a poor validity of MESS in predicting the need for amputation for high values of the score. It emerges from our study that, on the one hand, an attempt to rescue the limb is certainly mandatory in the case of low MESS values (<7), while on the other hand, the probability of amputation proportionally increases for MESS values equal or above 7.

**Figure 1:**
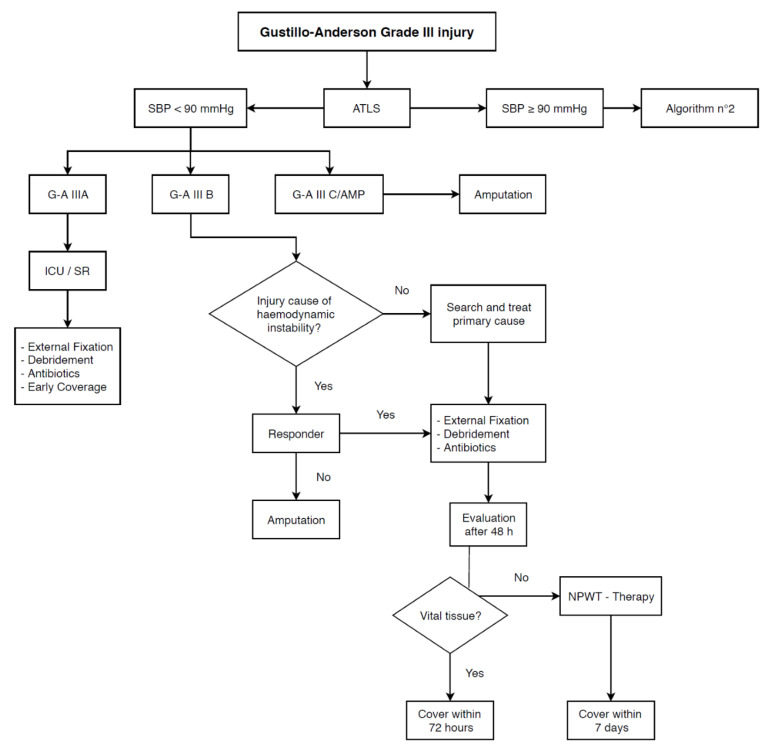
Algorithm no. 1.

**Figure 2:**
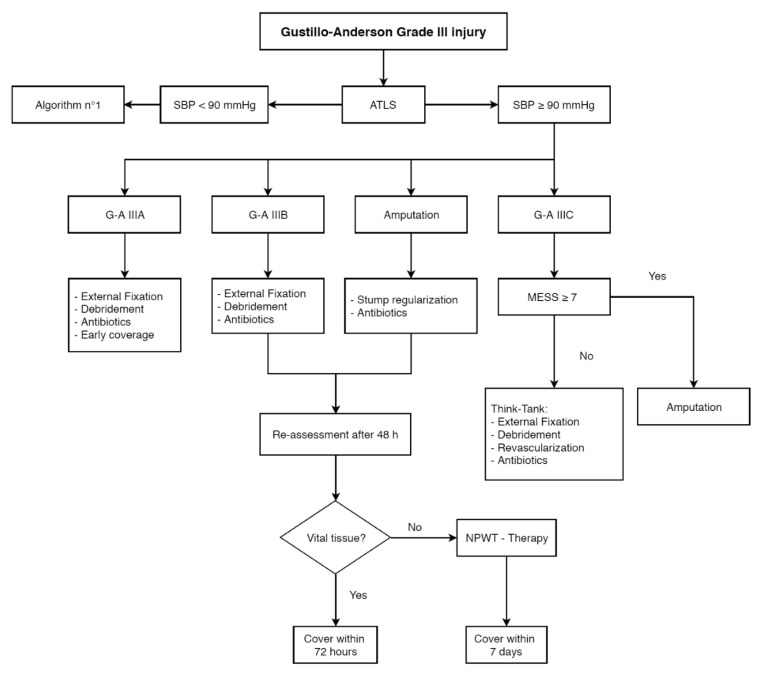
Algorithm no. 2.

MESS was the first developed score to assess the gravity of limb injury. Even if recent studies in literature do not emphasize its accuracy, it remains the most utilized score in clinical practice and its effectiveness should be reassessed within a therapeutic algorithm. Further prospective studies including MESS in our flowchart are needed to clarify its validity as a decision tool in complex mangled extremity management. Based on these results, we propose a management algorithm for high-grade limb open fractures. For patients presenting with hypotension (SBP < 90 mmHg) and a G/A IIIA, early debridement associated with skin coverage and adequate antibiotic therapy should be performed. In the case of G/A IIIB fractures, once other causes of hemodynamic instability are ruled out, patients who respond to adequate resuscitation and temporary hemostatic maneuvers (i.e., compression and tourniquet) should undergo external fixation associated with debridement and antibiotic therapy. Otherwise, in the case of G/A IIIB not responding to resuscitation or G/A IIIC/AMP, amputation is the recommended treatment option (Figure 1). For hemodynamically stable patients (SBP ≥ 90 mmHg) presenting with a G/A IIIC fracture, MESS guides further management: for scores below 7, external fixation, debridement, and antibiotic therapy should be considered. A multidisciplinary approach including a vascular surgeon to evaluate the possibility of revascularization of the limb must be considered. In contrast, for scores equal or above 7, amputation of the limb guarantees the best outcome (Figure 2). Regardless of the hemodynamics, systematic re-evaluation after 48 h from external fixation is mandatory to assess the viability of soft tissues. In the case of vital tissue, early coverage (within 72 h) is recommended; if not, as suggested by a recent consensus conference from the Milan Trauma Update 2017 edition, negative pressure wound therapy (NPWT) and delayed coverage (within 7 days) seem to be reasonable approaches [29,30].

Nevertheless, our study presents some limitations: first of which is its retrospective nature. Moreover, the sample size is quite small, and the subgroup analysis performed in order to identify possible predictors of limb amputation contributed to a further and significant reduction in the sample size. The limited number of events (amputations) forced us to enter only two variables into the multiple regression model. Therefore, the possibility to add other covariates in the model, which could have had a potential confounding or predicting effect on the outcome, was hindered, somehow reducing the power of our statistics. Moreover, the Hosmer–Lemeshow test failed to demonstrate an adequate goodness of fit for the model.

On the other hand, it is worth underlining how our data suggest that early coverage of damaged limb, after adequate debridement and lavage, seems to be the most powerful strategy to avoid infections, especially in mild and moderate injuries (A group). The perfect timing should be within 72 h from trauma and within a maximum of 7 days from the arrival in ED in the case of NPWT.

In conclusion, MESS and SBP have been demonstrated to be the two most important predictors of amputation and should be taken into consideration in structured management protocols. Referral to high-volume centers with specific expertise is mandatory for an adequate management of these patients and the achievement of best results.

## Figures and Tables

**Table 1:** Trauma-related data.

	A Group (26)		BC Group (66)		AMP Group (14)		Total (106)	
Variable	Number/Median	%/IQR	Number/Median	%/IQR	Number/Median	%/IQR	Number/Median	%/IQR
Age	37	27	46	23	43	24	43	23
Mechanism of Trauma								
Road Accident	16	61.5	38	57.6	8	57.1	62	58.5
Fall	6	23.1	9	13.6	0	0	15	14.2
Pedestrian	1	3.8	8	12.1	5	35.7	14	13.2
Crush	0	0	6	9.1	0	0	6	5.7
Penetrating	2	7.7	2	3	1	7.1	5	4.7
Cyclist	1	3.8	3	4.5	0	0	4	3.8
Shock Class								
I	15	57.7	30	45.5	3	21.4	48	45.3
II	6	23.1	16	24.2	3	21.4	25	23.6
III	3	11.5	10	15.2	5	35.7	18	17
IV	2	7.7	9	13.6	3	21.4	14	13.2
SBP in ED (mean)	120.36	25.67	118.81	35.4	105.31	38.88	117.44	33.73
HR in ED	92	23	91	35	115	39	92	33
GCS	15	0	15	1	15	11	15	1
LOS	24	23	40	33	37	39	36	32
ISS	14	18	13	19	19	27	14	19
Outcome								
Survived	24	92.3	59	89.4	10	71.4	93	87.7
Dead	2	7.7	7	10.6	4	28.6	13	12.3

SBP: Systolic Blood Pressure; HR: Heart Rate; ED: Emergency Department; LOS: Length of Stay; ISS: Injury Severity Score; TRISS: Trauma and Injury Severity Score.

**Table 2:** Comparisons among BC subgroups.

	BC-SALV (44)		BC-AMPT0 (11)		BC-AMPT1 (11)		
Variable	Number/Median	%/IQR	Number/Median	%/IQR	Number/Median	%/IQR	*p*
SBP (mean)	123.72	34.52	105.11	35.29	93.44	27.16	0.031 *
HR	91	26	100	27	100	27	n.s.
SC ≥ 3	12	25	4	44.4	4	44.4	n.s.
BE	−3.95	5.28	−3.3	4.3	−4.6	5.4	n.s.
Lac	3.15	2.43	2.85	2.55	2.78	1.9	n.s.
ICU-LOS (range)	3	9	3	9	14	26	0.04 *
H-LOS (range)	47.5	23	55	42	82	56	0.005 *
Head AIS ≥ 3	7	14.6	0	0	0	0	n.s.
Chest AIS ≥ 3	14	29.2	2	22.2	5	55.6	n.s.
Abdomen AIS ≥ 3	4	8.3	0	0	2	22.2	n.s.
G-A IIIC	10	22.7	8	72.7	8	72.7	<0.001 *
MESS	6	2	9	2	7	2	<0.001 *

SBP: Systolic Blood Pressure; HR: Heart Rate; BE: Base Excess; Lac: Lactate; ICU-LOS: Intensive Care Unit Length of Stay; H-LOS: Hospital Length of Stay; AIS: Abbreviated Injury Scale; ISS: Injury Severity Score; MESS: Mangled Extremity Severity Score. *: (*p* < 0.05).

## Data Availability

The data presented in this study are available on request from the corresponding author. The data are not publicly available to preserve confidentiality.
